# Real and Perceived Risks for Mycotoxin Contamination in Foods and Feeds: Challenges for Food Safety Control

**DOI:** 10.3390/toxins2040572

**Published:** 2010-04-01

**Authors:** Dragan R. Milićević, Marija Škrinjar, Tatjana Baltić

**Affiliations:** 1 Institute of Meat Hygiene and Technology, Kaćanskog 13, 11000 Belgrade, Serbia; Email: tanja@inmesbgd.com (T.B.); 2 Faculty of Technology, Bulevar Cara Lazara 1, 21000 Novi Sad, Serbia; Email: skrinjarm@uns.ac.rs (M.Š.)

**Keywords:** mycotoxins, food safety, human and animal health, risk analysis

## Abstract

Mycotoxins are toxic compounds, produced by the secondary metabolism of toxigenic moulds in the *Aspergillus*, *Alternaria*, *Claviceps*, *Fusarium*, *Penicillium* and *Stachybotrys* genera occurring in food and feed commodities both pre- and post-harvest. Adverse human health effects from the consumption of mycotoxins have occurred for many centuries. When ingested, mycotoxins may cause a mycotoxicosis which can result in an acute or chronic disease episode. Chronic conditions have a much greater impact, numerically, on human health in general, and induce diverse and powerful toxic effects in test systems: some are carcinogenic, mutagenic, teratogenic, estrogenic, hemorrhagic, immunotoxic, nephrotoxic, hepatotoxic, dermotoxic and neurotoxic. Although mycotoxin contamination of agricultural products still occurs in the developed world, the application of modern agricultural practices and the presence of a legislatively regulated food processing and marketing system have greatly reduced mycotoxin exposure in these populations. However, in developing countries, where climatic and crop storage conditions are frequently conducive to fungal growth and mycotoxin production, much of the population relies on subsistence farming or on unregulated local markets. Therefore both producers and governmental control authorities are directing their efforts toward the implementation of a correct and reliable evaluation of the real status of contamination of a lot of food commodity and, consequently, of the impact of mycotoxins on human and animal health.

## 1. A Widespread Problem

Mycotoxins are a structurally diverse group of mostly small molecular weight compounds, produced by the secondary metabolism of some filamentous fungi or moulds [1,2], which under suitable temperature and humidity conditions, may develop on various foods and feeds, causing serious risks for human and animal health [3,4]. In terms of structural complexity, mycotoxins vary from simple C_4 _compounds, e.g., moniliformin, to complex substances such as the phomopsins [5] and the tremorgenic mycotoxins [6]. Although currently more than 300 mycotoxins are known, scientific attention is focused mainly on those that have proven to be carcinogenic and/or toxic. Human exposure to mycotoxins may result from consumption of plant-derived foods that are contaminated with toxins, the carryover of mycotoxins and their metabolites in animal products such as meat and eggs [7] or exposure to air and dust containing toxins [8]. During the last 15–20 years, case studies have shown that people living and working in damp or moldy buildings have an increased risk of adverse health effects including impaired immune function, bronchitis, asthma, recurrent airway infections, and extreme fatigue [9]. More recently, the presence of *Stachybotrys chartarum* has been associated with the development of idiopathic pulmonary hemosiderosis (IPH) in infants, although the active toxins and mechanisms of exposure remain unclear [10]. 

Human food can be contaminated with mycotoxins at various stages in the food chain [11,12] and the most important genera of mycotoxigenic fungi are *Aspergillus*, *Alternaria*, *Claviceps*, *Fusarium*, *Penicillium* and *Stachybotrys.* The principal classes of mycotoxins include a metabolite of *Aspergillus flavus* and *Aspergillus parasiticus*, aflatoxin B_1 _(AFB_1_), the most potent hepatocarcinogenic substance known, which has been recently proven to also be genotoxic. In dairy cattle, another problem arises from the transformation of AFB_1 _and AFB_2 _into hydroxylated metabolites, aflatoxin M_1 _and M_2 _(AFM_1 _and AFM_2_), which are found in milk and milk products obtained from livestock that have ingested contaminated feed [13]. Ochratoxin A (OTA) is a secondary metabolite produced by several species of *Aspergillus* and *Penicillium* fungi. The toxin, which is a nephrotoxic and nephrocarcinogenic compound, has mainly been found in cereals as well as in other products like coffee, wine, dried fruits, beer and grape juice. It occurs in the kidney, liver and blood of farm animals by transfer from animal feed. Although its genotoxic power has so far not been definitively established, zearalenone (ZEA), produced by various species of *Fusarium*, in particular *F. graminearum* and *F. culmorum*, has an estrogenous action and is significantly toxic to the reproductive system of animals. 

The trichothecenes (TCT), are a family of 200–300 related cyclic sesquiterpenoids produced by *Fusarium*, *Myrotecium*, *Trichoderma*, *Cephalosporium*, *Verticimonosporium*, and *Stachybotrys* species, which are divided into four groups (types A–D) according to their characteristic functional groups. Type-A and –B trichothecenes are the most common. Type A is represented by HT-2 toxin and T-2 toxin and type B is most frequently represented by DON, 3-acetyl-DON (3-Ac-DON), 15-acetyl-DON (15-Ac-DON), nivalenol (NIV), and fusarenon X (FUS-X). Type C and D trichothecenes are characterized by a second epoxide (C-7,8 or C-9,10) or an ester-linked macrocycle (C-4,16), respectively, and are not associated with Fusarium head blight (FHB). The toxic effects of trichothecenes include gastrointestinal effects such as vomiting, diarrhea and bowel inflammation. Anemia, leukopenia, skin irritation, feed refusal and abortion are also common. The trichothecenes as a group are immunosuppressive. The Fumonisins is (FUM) family of mycotoxins produced by the species of *Fusarium* in the Liseola section. *F. verticilloides* (formerly *F. moniliforme*), a species that is almost ubiquitous in corn, and *F. proliferatum* are the main species producing high yields of FUM. Fumonisins B1, B2 and B3 (FB1, FB2 and FB3) occur in fungal cultures or are found in naturally contaminated corn samples. FUM are structurally similar to sphingosine, a component of sphingolipids. Sphingolipids are in high concentrations in myelin and in certain nerve tissues. FUM toxicity is thought to result from blockage of sphingolipid biosynthesis. FUM has been shown to cause leucoencephalomalacia in horses (ELEM), pulmonary edema in swine (PPE) and hepatoxicity in rats [14].

In the mid 1980s, the topic of conjugated or masked mycotoxins received attention, because in some cases of mycotoxicoses, clinical observations in animals did not correlate with the low mycotoxin content determined in the corresponding feed. The unexpected high toxicity was attributed to undetected, conjugated forms of mycotoxins that hydrolyze the precursor toxins in the digestive tract of animals. As part of their metabolism, plants are capable of transforming mycotoxins into conjugated forms [15,16,17]. So far, natural occurrence of a zearalenone glucoside [18] and deoxynivalenol glucoside [16,17,18] have been reported. Gareis [19] demonstrated that zearalenone-4-beta-d-glucopyranoside was decomposed during digestion, releasing zearalenone into the animal gut. As zearalenone-glycoside is not detected during routine analysis but is hydrolyzed during digestion, it seems likely that masked mycotoxins may contribute to cases of mycotoxicoses. High-performance liquid chromatography (HPLC) combined with tandem mass spectrometry (MS/MS) offers a powerful tool for identification and characterization of mycotoxin conjugates [15]. 

The impact of mycotoxins on health depends on the concentration and duration of exposure to the toxin, the toxicity of the compound, e.g., acute or chronic (e.g., carcinogenic) effects, the body weight of the individual, the presence of other mycotoxins (synergistic effects), environmental factors (farm management) and other dietary effects [20,21]. The incidence and extent of mycotoxin contamination is strictly related to geographic and seasonal factors as well as cultivation, harvesting, stocking, and transport conditions [22]. The evaluation of the incidence and extent of contamination of foodstuffs is crucial and has, in fact, been taken into account for many years by the various disciplines that concur in the definition and management of the risk associated with these toxins and its management [23].

## 2. Mycotoxin Occurrence and the Implications for Human and Animal Health

A wide range of commodities can be contaminated with mycotoxins both pre- and post-harvest [7]. Aflatoxins (AFTs) are found in maize and peanuts, as well as in tree nuts and dried fruits. OTA is found mainly in cereals, but significant levels of contamination may also occur in wine, coffee, spices and dried fruits. Other products of concern are beans, roasted coffee and cocoa, malt and beer, bread and bakery products, wines and grape juices, spices, poultry meat and kidneys, pig kidneys and pork sausages [24,25,26,27,28,29,30]. OTA and AFB_1_ are among the most frequently observed combinations of mycotoxins in different plant products [31]. Most mycotoxins are relatively heat-stable within the range of conventional food processing temperatures (80–121 °C), so little or no destruction occurs under normal cooking conditions such as boiling and frying, or even following pasteurization. In fact, during the production of cheese and other types of milk products the amount of AFM_1_ may increase (depending on the type of cheese). Available evidence suggests that tissue accumulation of these mycotoxins, or their metabolites, is very low and is excreted into milk from 1–6% of dietary intake [13,32,33]. Residues of cyclopiazonic acid (CPA), a co-contaminant with aflatoxin, have been found in meat, milk and eggs [34]. 

Fusariotoxins are commonly found in cereals and their products, which constitute an important part of human food and animal feed. Highly contaminated crops are frequently directed to animal feed [35,36,37]. ZEA is a secondary fungal metabolite produced by several species of *Fusarium* fungi, mainly by *F. graminearum* and *F. culmorum*. These species are known to colonize maize, barley, oats, wheat, and sorghum, and tend to develop during prolonged cool, wet growing and harvest seasons in the temperate and warm regions of the world. Main animal and human exposure comes from chronic contaminated food ingestion but human exposure can be direct *via* cereals or indirect *via* animal products. As fusariotoxins are hydrosoluble, they are generally weakly accumulated in animal tissues [38]. Consequently, the possible presence of toxic residue in animal products (milk, meat, *etc.*) remains unclear. Trichothecenes are rapidly and dramatically eliminated without accumulation in organisms [39]. Only compound traces could still be detected 24 h after oral exposure. After an extensive review of the literature, Pestka [40] concluded that trace levels of mycotoxins and their metabolites may carry over into the edible tissue (meat) of food producing animals. 

Although most reports of mycotoxicoses have been associated with ingestion of infected food, there is evidence that airborne mycotoxins from indoor molds can also produce human illness, most likely caused by inhalation [44]. Health concerns relating to indoor molds have dramatically risen over the past decade. Indoor molds are perceived by many to be a growing problem in the USA, Canada and Europe [41,42,43]. Case studies have shown that people living and working in damp or moldy buildings suffer from very similar vague complaints such as airway infections, impaired immune function, bronchitis, asthma, irritation of eyes and extreme fatigue. These effects have come to be called ‘Sick Building Syndrome’, which means that no specific etiological factor can be identified. Molds growing on building materials can be divided into three groups based on their water activity (aw), requirements on laboratory substrates, and responses to changes in aw [45]. Primary colonizers or storage molds, capable of growing at a_w_ < 0.8 (many with optimal growth rates at a_w_) approaching *Penicillium chrysogenum* and *Aspergillus versicolor* are the most common species. Secondary colonizers or phylloplane fungi, requiring a minimal a_w_ between 0.8 and 0.9. This group comprises species of *Alternaria*, *Cladosporium*, *Phoma*, and *Ulocladium*. These are able to thrive under conditions where marked changes in humidity occur during the day. Tertiary colonizers or water-damage molds, needing a_w_ > 0.9, include many of the most toxic species such as *Chaetomium* *globosum*, *Memnoniella echinata*, *Stachybotrys chartarum*, and species of *Trichoderma*. Several of these are considered tropical fungi, which seems consistent with their propensity for growth in humid buildings. Studies concerning indoor health problems and mycotoxins mainly focus on trichothecenes. It has been shown that isolates from fungi-damaged buildings produce highly cytotoxic trichothecenes (verrucarins, roridins, verrucarols, T-2 toxin, diacetoxyscirpenol, nivalenol and deoxynivalenol), but also ochratoxin A, zearalenone, aflatoxins and sterigmatocystin [9,35,46,47,48].

In general, mycotoxin exposure is more likely to occur in parts of the world where poor methods of food handling and storage are common, where malnutrition is a problem, and where few regulations exist to protect exposed populations. However, even in developed countries, specific subgroups may be vulnerable to mycotoxin exposure. In the United States, for example, Hispanic populations consume more corn products than the rest of the population, and inner city populations are more likely to live in buildings that harbor high levels of molds 49].

Although hundreds of mycotoxins have been identified, information about many of them is limited with regard to their natural occurrence, stability in foods and feeds, and toxicity to humans and animals. In view of the diversity of toxicological manifestations and the economic losses after exposure to certain mycotoxins, there is a continuous need to protect the health of humans and susceptible animals by limiting their exposure to these toxins. 

AFB_1_ has been extensively linked to human primary liver cancer (PLC) [50], in which it acts synergistically with hepatitis B virus (HBV) infection and was classified by the International Agency for Research on Cancer (IARC) as a human carcinogen (group 1 carcinogen) [51]. This combination represents a heavy cancer burden in developing countries. Hepatocellular carcinoma (HCC) is the sixth most common cancer worldwide with 626,000 new cases in 2002 (5.7% of the total) [52]. Mortality is almost synonymous with incidence, given the poor survival rates. In 2002 there were 598,000 deaths in the world from HCC. The majority of HCC cases (>80%) occur in developing countries with a higher incidence in males than females. In Europe, the incidence rates for HCC are generally low. Given recently published liver cancer incidence rates in the European Union of 10.0 per 100,000 males and 3.3 per 100,000 females [53], it is clear that aflatoxin plays a significant role in liver cancer in developing countries, but not in the developed world where other risk factors such as cirrhosis are more important. AFM_1_, according to the classification of IARC [51], was first classified in the second group of carcinogens (2B), but in the year 2002 [50], the same organization classified it in the first group of carcinogens. AFB_1_, however is a genotoxic, carcinogen and the safety factors used for non-genotoxic carcinogens cannot apply. Therefore, most agencies have not set a Tolerable Daily Intake (TDI) for AFB_1_. AFB_1_ is moreover considered the main hepatocarcinogen in animals, although effects vary with species, age, sex, and general nutritional conditions. Trout, ducklings and pigs are highly susceptible, with ruminants being less susceptible [54]. Fumonisin B_1_ (FB_1_) is always the most abundant and toxic metabolite of this group of mycotoxins, representing ca. 70% of the total concentration in naturally contaminated foods and feeds, followed by FB_2_ and B_3_. FUM 's are structurally similar to sphingolipids and their bases, and they inhibit ceramide syntethase, an enzyme in their biosynthetic pathway. This inhibition results in increased levels of sphingoid bases (sphinganine and sphingosine) in serum of exposed animals. FUM have been implicated in one incident of acute food-borne disease in India in which the occurrence of borborygmy, abdominal pain, and diarrhea was associated with the consumption of maize and sorghum contaminated with high levels of FUM [55]. FB_1_, the most abundant of the numerous FUM analogs, was classified by the IARC as a group 2B carcinogen (possibly carcinogenic in humans) [53]. Studies in the former Transkei region of South Africa and in Linxian and Cixian counties, China, have demonstrated an association between FUM exposure in rural subsistence farming areas and a high incidence of esophageal cancer as well as with field outbreaks of ELEM in many countries such as Egypt, South Africa and the United States of America [56] and pulmonary edema in swine (PPE) [57,58]. ELEM is a fatal neurological disease of horses, characterized by liquefactive necrosis of the white matter of the brain. ELEM has been experimentally induced in horses by either supplementing their diets with *F. moniliforme-*contaminated corn, or by the oral administration of FB_1_, a toxin produced by *F. moniliforme* [59]. FUMs, which inhibit the uptake of folic acid *via* the folate receptor [60], have also been implicated in the high incidence of neural tube defects in rural populations known to consume contaminated maize, such as the former Transkei region of South Africa and areas of northern China [61]. Besides their hepatotoxicity [62] and nephrotoxicity [63], they affect also the immune system [64,65]. Risk assessments for FUMs have been performed by the Scientific Committee on Food (SCF) [66], the Joint FAO/WHO Expert Committee on Food Additives (JECFA) [67] and a Nordic Working Group [68]. A group provisional maximum tolerable daily intake (PMTDI) for FB_1_, FB_2_ and FB_3_ of 2 mg/kg body weight was established by JECFA. A group TDI of 2mg/kg body weight was also established by SCF [66]. The tolerable intake established by JECFA and SCF was based on a no-observed-adverse effect level (NOAEL) of 0.2 mg FB1/kg body weight/day for kidney toxicity in the rat, the most sensitive adverse effect observed. The presence of other fusariotoxins, such as moniliformin or culmorin, is only rarely reported so they will not be evoked here. 

The other three agriculturally important mycotoxins have also been associated with various outbreaks of human disease, mostly in developing countries. A number of occurrences of acute food-borne illness in India and China involving gastrointestinal symptoms have been attributed to the consumption of deoxynivalenol (DON)-contaminated cereals [69,70]. TCT are responsible for a wide range of disorders in animals, including feed refusal, weight loss and vomiting. An extensive toxicological evaluation of TCT in animal feed was conducted by Eriksen and Pettersson [71]. In particular, DON inhibits protein biosynthesis and has been reported to suppress immune responses [72]. Higher intakes than the recommended TDI (1 mg kg ^-1^ bodyweight day ^-1^) were recognized, especially for infants and children, and indicated clearly that the presence of T-2 and HT-2 should be of concern for public health [73]. OTA has long been associated with Balkan endemic nephropathy (BEN), a fatal renal disease with histopathological similarities to OTA-induced nephropathy in swine and has been associated with incidences of epithelial tumors of the upper urinary tract [74,75]. OTA was classified by the IARC as possibly carcinogenic to humans (group 2B carcinogen) [76]. The European legislation estimated the TDI for OTA at 5.8 ng OTA kg^-1^ body weight per day [77]. However, as there are differences in food intake (per unit of body weight) for children, adolescents and adults, for a given concentration of a contaminant, a child or an adolescent will receive different exposure than an adult. The only estrogenic mycotoxins yet established are the zearalenones, although there is no reason to believe that other structural classes will not be discovered in the future. ZEA is a naturally occurring endocrine-disrupting chemical and has been associated with clinical manifestations of various estrogenic effects in humans and farm animals, especially pigs [78], including an outbreak of precocious pubertal changes in young children in Puerto Rico in the Caribbean [79] and gynecomastia with testicular atrophy in rural males in southern Africa [80]. ZEA is, therefore, a frequently analyzed mycotoxin, and analysis of ZEA has been included in the internal quality control of maize production by many cereal handling companies. Besides sampling, the major problems for regulation and control of the ZEA content have so far been the lack of fast analytical methods and a lack of comparability of measurement results and of appropriate reference materials.

Various mycotoxins may occur simultaneously, depending on the environmental and substrate conditions [81]. Considering this coincident production, it is very likely that humans and animals are exposed to mixtures rather than to individual compounds. The interactive (synergistic) cytotoxic effects of OTA, Ochratoxin B (OTB), citrinin, and patulin, which are produced by a number of *Penicillium* and *Aspergillus* species, was recently evaluated by Heussner *et al.* [82]. By application of a step-wise approach to test combination toxicity, using various full factorial as well as a central composite experimental design, the interactive (synergistic) cytotoxic effects of the these four toxins were assessed. These findings indicate that the toxicity of mycotoxin mixtures cannot be accurately predicted only on the basis of the effect of the individual toxins. These aspects must be considered in future risk assessment studies. 

The evaluation of mycotoxins in biological fluids can provide useful indications of the dietary intake of mycotoxins. This approach can also constitute a valid, although indirect, evaluation of mycotoxin contamination in foodstuffs. This methodology can somehow give a better estimate of the exposure of humans to mycotoxins than that obtained by monitoring food data since the latter may, as stated, be affected by sampling, subsampling, and analysis errors. 

## 3. The Importance of Mycotoxin Control

Mycotoxins are secondary metabolites of fungi. It is not possible to predict their presence or to prevent their occurrence during preharvest, storage, and processing operations by current agronomic practices. Therefore, their presence in food and feed represents a constant health risk for animals and humans. Regulations relating to mycotoxins have been established in many countries to protect the consumer from the harmful effects of these compounds. In several countries, these contaminants are subject to legislation that is based on the establishment of an Acceptable daily intake (ADI) or Tolerable daily intake (TDI). Different factors play a role in the decision-making process of setting limits for mycotoxins. These include:

- the availability of toxicological data on mycotoxins,
- the availability of exposure data on mycotoxins,
- knowledge of the distribution of mycotoxin concentrations within commodity or product lots,
- the availability of analytical methods,
- legislation in other countries with which trade contacts exist,
- the need for sufficient food supply.

**Figure 1:**
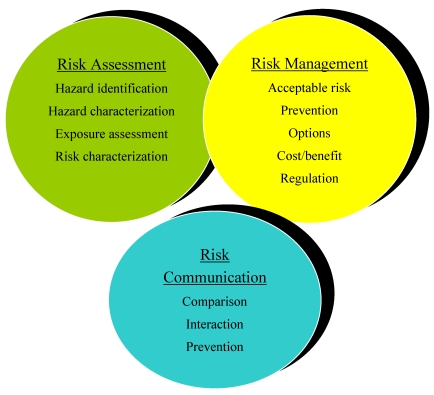
Risk analysis framework for food safety.

The first two factors provide the information necessary for hazard assessment and exposure assessment, respectively; the main bases of risk assessment. Risk assessment is the scientific evaluation of the probability of the occurrence of known or potential adverse health effects resulting from human exposure to food-borne hazards. It is the primary scientific basis for promulgation of regulations. The third and fourth factors are important for enabling practical enforcement of mycotoxin regulations through adequate sampling and analysis procedures. The last two factors are merely socio-economic in nature, but are equally important in the decision-making process to establish meaningful regulations and limits for mycotoxins in food and feed. Risk assessment regulations are primarily based on known toxic effects. For the mycotoxins currently considered most significant (AFB_1_, B_2_, G_1 _and G_2_; AFM_1_; OTA; patulin; FB_1_, FB_2, _and FB_3_, ZEA, T-2, HT-2 toxins and DON), the Joint Expert Committee on Food Additives (JECFA-Scientific Advisory Body of the World Health Organization WHO) and the Food and Agriculture Organization (FAO) has evaluated their hazard in several sessions [83,84,85]. In February 2001, a special JECFA session was devoted to emergency mycotoxins. Two reports have appeared following this session: a longer version [86] and a shorter version [87]. These reports provide good and detailed insight into the process of risk assessment of mycotoxins. The reports addressed several concerns about the mycotoxins considered - their properties and metabolism, toxicological studies, and final risk evaluation. With the mycotoxin evaluations, the Committee discussed general considerations on sampling, analytical methods associated intake issues and control. Risks associated with mycotoxins depend on both hazard and exposure. The hazard of mycotoxins to individuals is probably, more or less, the same all over the world (although other factors are, sometimes, also important, e.g., hepatitis B virus infection in relation to the hazard of aflatoxins). Exposure is not the same due to different levels of contamination and dietary habits in various parts of the world. The risk analysis framework for food safety is illustrated in Figure 1.

## 4. Legislative Frameworks on Analytical Methods

The most prominent reason for collecting food samples for the investigation of contaminants such as mycotoxins, is to protect consumer health, mainly by verifying the compliance of food and feed with acceptable safety standards. The correct evaluation of mycotoxin contamination in foodstuffs depends principally on the degree of accuracy associated with the individual steps by which this information is obtained. Because the distribution of mycotoxins is often highly heterogeneous, taking a representative sample is the most critical stage. Thus, the error associated with sampling procedures is notably higher than that associated with subsampling or analysis. 

Sampling is one of the most crucial, but underestimated parts of the multifaceted and complex bulk of activities aimed at addressing and managing food issues. In practice, the overall objective of good sampling is to provide reliable samples to be analyzed that can represent the basis for ‘‘fit for purpose’’ investigations. In most cases, meaningful sampling is a process comprising two very dissimilar steps

(1) The first step (hereafter referred to as ‘‘primary sampling’’) consists of taking the decision on “why, where and when” to collect the samples. In other words, the process of “statistically” locating the sites (populations) from which food samples should be taken;
(2) The second step (hereafter referred to as “secondary sampling”) consists of establishing how samples should be collected in order to be representative of the lot under investigation. For both steps, the quality and the consequent reliability of the data are strongly dependent on the available resources and on the skill of the people involved.

For this class of contaminants, the need for statistically-based planning is particularly relevant for: (i) The multifaceted implications of mycotoxin contamination (health, trade, ethical issues related to developing countries’ difficulties), and (ii) the largely non-homogeneous distribution of the toxins within food commodities, with the consequent need for careful secondary sampling. Appropriate sampling plans are essential to ensure that the analytically-derived mean concentration of a sample is representative of the true mean concentration of a lot. Sampling plans are particularly relevant in the area of mycotoxins, where it is known that the contamination of a commodity can be heterogeneously distributed. Good primary sampling schemes have so far been developed for several classes of contaminants, such as dioxins and pesticides [88], in contrast to the very few valid ones so far proposed for mycotoxins. In contrast, a large number of papers have appeared related to secondary sampling schemes for AFB_1_ (particularly on its distribution in a lot and on related sampling plans) [89], but only a few studies deal with some *Fusarium* toxins [90,91]. Conversely, specific studies focused on the distribution of OTA-contaminated units are not yet available, apart from the vague assumption that ‘‘representative sampling’’ for aflatoxins is more difficult than sampling for other known mycotoxins in food products. Sampling procedures recommended for aflatoxins should thus be adequate for other mycotoxins [92]. Nevertheless, the European legislation dealing with sampling and methods of analysis of mycotoxins for official control was recently adopted [93]. 

In conclusion, the analysis of sources of errors in evaluating the impact of mycotoxins on human health should be carefully performed taking into account many aspects such as planning and accomplishment of monitoring programs, consumer’s health protection, economic, political and commercial considerations. 

### State-of-the-art analytical methods

**Figure 2:**
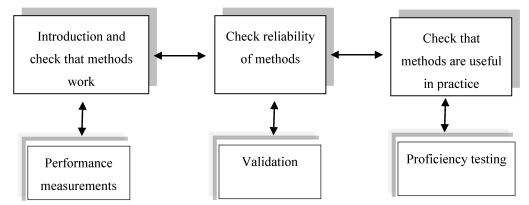
Performance measurement, validation, and proficiency testing (Adapted from 98).

Legislation calls for monitoring methods. Reliable analytical methods must be available to enable enforcement of the regulations in daily practice. In addition to reliability, simplicity is desired, as it will affect the amount of data generated and the practicality of the ultimate measures taken. The reliability of mycotoxin analysis data can be improved by use of interlaboratory-validated methods of analysis (e.g., the methods of Association of Official Agricultural Chemists (AOAC) International and methods standardized by European Standardization Committee-CEN) (Figure 2). These methods have been largely developed in response to planned regulations for mycotoxins or regulations that came into force. The requirements for these methods were dictated by the needs, *i.e.*, they had to be suitable for the (planned) regulated mycotoxin–matrix combination(s). The limits of determination of the methods had to be demonstrated to be low enough for precise and accurate determination of the mycotoxins of interest at regulatory levels. Methods were also developed and validated for toxin–matrix combinations for which there were no regulations (yet), but for which the scientific community saw a need, e.g., for surveillance purposes. These developments eased the establishment of specific mycotoxin regulations. AOAC currently has approximately 45 analytical methods for determination of mycotoxins [94]. All have undergone extensive testing in interlaboratory validation studies, and subsequent review by the AOAC’s rigorous approval process. AOAC methods are referred to as official methods in mycotoxin legislation in a few dozen countries [95]. In Europe, CEN methods are becoming increasingly important. Ten mycotoxin methods have been standardized by the CEN, and this number will grow substantially in the years to come. Although CEN mycotoxin methods are not mandatory for official food control in the European Union (EU), all CEN mycotoxin methods can be used in the EU for official food-control purposes because their performance characteristics fulfill the criteria laid down in the EU regulation for sampling and analysis [96]. One of them, HPLC with different detectors, is frequently used both for routine analyses and as a confirmatory method for novel or screening techniques. For some mycotoxins, e.g., TCT, gas chromatography (GC) is the method more often used [97]. Except for direct mass spectrometric methods, all the other analytical methods used for mycotoxin determination are either immunoassay based, or otherwise fall into the category of direct or indirect screening methods. The use of good, validated methods of analysis is no guarantee that reliable analytical results will be obtained in mycotoxin determination. Analytical quality assurance (AQA) is another prerequisite for adequate food-law enforcement. AQA includes, where possible, the use of (certified) reference materials (e.g., CRMs supplied by the irmm.jrc.be). European Commission’s Joint Research Centre/Institute for Reference Materials and Measurements (JRC/IRMM). 

## 5. The International Mycotoxin Regulatory Situation

Mycotoxins may be present in many foods, feeds and commodities as a result of growth of mould on crops and foods, sometimes in quantities below the limit of detection of the analytical methods of today. As foods, feeds and raw materials and ingredients for food production are to an increasing extent traded across borders, there is an evident need for international legislation on mycotoxins in foods and feeds in order to avoid trade barriers and to protect the health of the consumer. Since the discovery of the AFT in 1960 and subsequent recognition that mycotoxins are of significant health concern to both humans and animals, regulations gradually developed for mycotoxins in food and feed. In the early days of mycotoxin regulations these measures focused mainly on the aflatoxins. They were established by industrialized countries and limits often had an advisory or guideline character. Over the years, the number of countries with known specific mycotoxin regulations has increased from 33 in 1981 [99] to 56 in 1987 [32], 77 in 1995 [100], and 100 in 2003 [95].

International legislation on foods and feeds is established by Codex Alimentarius (CAC). The Codex Alimentarius system for development of legislation concerning contaminants, including mycotoxins in foods and feeds, is laid down in considerable detail [101,102]. The Codex Committee on Food Additives (CCFAC) serves as the body responsible for the risk management component of the Codex Alimentarius risk analysis process in relation to contaminants in general and mycotoxins in foods and feeds in particular. The body responsible for the risk assessment component of the Codex Alimentarius risk analysis process is JECFA. It is the role and privilege of JECFA to provide Codex Alimentarius with scientifically based assessment of the toxicity of food additives and contaminants, such as mycotoxins, and to establish safe levels for human consumption. Hence, the 56th JECFA in February 2001 assessed several mycotoxins [86]. The Codex General Standards for Contaminants and Toxins in Food (GSCTF) covers also feeds and raw commodities. The GSCTF contains the most important principles for laying down Codex Maximum Limits (MLs) for contaminants and toxins in foods and feeds [103].

Current regulations encompass 13 different mycotoxins or groups of mycotoxins and specific limits have been established for many food and feed commodities and products. Until the late 1990s setting of mycotoxin regulations was mostly a national affair. Gradually, several economic communities e.g., EU, European Union; MERCOSUR Mercado Cómứn del Sur; Australia and New Zealand harmonized their mycotoxin regulations, thereby overruling existing national regulations. Current regulations are increasingly based on scientific opinions of authoritative bodies, for example the FAO/WHO Joint Expert Committee on Food Additives of the United Nations (JECFA) and the European Food Safety Authority (EFSA). At the same time, requirements for adequate sampling and analytical methods put high demands on other professional organizations, for example AOAC International and the European Standardization Committee (CEN). 

## 6. Economic Impact

Mycotoxin contamination of the food chain has a major economic impact. However, the insidious nature of many mycotoxicoses makes it difficult to estimate incidence and cost [104]. In addition to crop losses and reduced animal productivity, costs are derived from the efforts made by producers and distributors to counteract their initial loss, the cost of improved technologies for production, storage and transport, the cost of analytical testing, especially as detection or regulations become more stringent, and the development of sampling plans [91]. Although in Europe there are no data on the economic costs of mycotoxins, for only Hungary the direct and indirect losses were estimated in a wheat epidemic (1998) to 100 million Euro. In the United States alone, the mean economic annual costs of farmer gate cereal crop losses due to aflatoxins, fumonisins and trichothecenes, are estimated to be $932 million. There is also a considerable cost to society as a whole, in terms of monitoring extra handling and distribution costs, increased processing costs and loss of consumer confidence in the safety of food products. It is estimated that in developing countries, the greatest economic impact is associated with human health [105]. 

Delineating economic impact reflects the complexity of a mycotoxin contamination within the food chain. There is a clear need to protect consumers through regulations, but at what cost? A comprehensive risk and economic analysis of lowering the acceptable levels for FUM and AFT in world trade demonstrated that the United States would experience significant economic losses from tighter controls [106]. The developing countries, China and Argentina, were more likely to experience greater economic losses than sub-Sahara Africa. The disturbing outcome of this detailed analysis was that tighter controls were unlikely to decrease health risks and may have the opposite effect [106]. In other words, very stringent international trade regulations could lead to the situation where exporting countries, especially developing countries, would retain higher risk commodities which would subsequently be available for their own populations; communities which are already exposed to higher levels of mycotoxins than consumers in developed countries. 

## 7. Strategies to Prevent Mycotoxin Contamination of Food and Animal Feed

Many strategies to prevent mycotoxin contamination of food and animal feed have been developed [107,108]. It is clear that mycotoxins can contaminate agricultural produce, both in the field as well as during storage. The use of pre-harvest control strategies for such resistance varieties, field management, the use of biological and chemical agents, harvest management and post-harvest applications, including improving drying and storage conditions, together with the use of natural and chemical agents and irradiation have clearly been shown to be important in the prevention of mycotoxigenic mould growth and mycotoxin formation [109]. The importance of drying and moisture control during storage is generally well understood by the industry, in terms of the importance of prevention of fungal contamination. Interesting results have been reported on the potential use of biocompetitive agents in different biological control strategies to prevent the pre-harvest aflatoxin contamination of crops, such as peanuts, rice, maize, and cottonseed. It is clear that much more work must be conducted to identify various crop genotypes which are resistant to mycotoxigenic fungus infection and subsequently mycotoxin formation. It is also clear that a combination of the development of crop species with resistance to toxigenic fungi and biocompetitive non-mycotoxigenic strain technologies may yield one of the most effective strategies for prevention of mycotoxin contamination [110,111]. Several natural plant extract and spice oils of eugenol, cinnamon, oregano, onions, lemongrass [112,113], tumeric, mint, and chemical compounds (fungicide, herbicide, and surfectant) are known to prevent both mycotoxigenic mould growth and mycotoxin formation during post-harvest season. In addition to application of plant extracts and chemical agents as well, sodium bisulfite, and chlorine seem to hold great potential in the detoxification of mycotoxins, unfortunately their use significantly decreases the nutritional value of the foods or produces toxic derivatives in the treated product with undesirable sensory properties. This will severely limit their widespread use. At the same time it should be noted that chemical treatment is not allowed within the EC for commodities destined for human consumption. Recently, there has been an increasing interest in the use of bacteria, yeast, and fungi to help reduce the toxic effect of mycotoxins [114]. While most studies to date on mycotoxin detoxification by microorganisms have been undertaken under laboratory conditions, there is data on the effective use of *F. aurantiacum* in the detoxifying AFB as antagonistic microorganisms, such as lactic acid bacteria with their antifungal properties, seem to be potentially very effective in the prevention of mycotoxin formation. The precise antifungal properties of lactic acid bacteria are still largely unresolved but may involve microbial competition [115], as well as extracellular metabolites which are heat-stable and of low molecular weight. Again, further investigations are clearly needed to gain a better understanding of this antifungal action. Various physical and chemical strategies have also been developed to help prevent mycotoxin contamination, including physical separation, extraction with sorbents, and adsorption [116]. The fluorescence sorting of maize, cottonseed and figs by examination under UV light is known to be the cheapest and the simplest acceptable way for the screening of aflatoxins. It is clear that no single currently available physical or chemical detoxification method will be suitable for all foods and animal feeds. The effectiveness of a method in the detoxification of mycotoxins depends on the nature of the food, environmental conditions such as moisture content, temperature, as well as the type of mycotoxin, its concentration and the extent of binding between mycotoxin and constituents. While a range of chemical compounds, including hydrochloric acid, ammonia, hydrogen peroxide, ozone,from various food products, including milk, peanuts, maize, and red pepper without leaving toxic end products. One potential drawback here is the production of a bright orange pigment by the organisms which restricts its use in the detoxification of food and in feed fermentations. The most recent approach to the problem has been the use of mycotoxin-binding agents in the diet that sequester the mycotoxin in the gastrointestinal tract thus reducing their bioavailability. Although activated charcoal (AC), hydrated sodium calcium aluminosilicate (HSCAS), aluminosilicate, zeolite, and bentonite have shown good potential for use in the animal feed to help overcome aflatoxicosis, the future *in vivo* investigations must focus on other problematic mycotoxins. Interestingly lactic acid bacteria and bifidobacteria have been shown to bind AFB_1_, but mechanistic studies need to be conducted on the precise binding mechanism, while the conditions favoring the release of bound toxin molecules need to be investigated as well. 

## 8. Conclusions

Toxic levels of many naturally occurring toxins are often produced only under certain environmental conditions. Identification and prevention of these environmental conditions will play an important role in minimizing the adverse effects of these toxins. However, because many of these environmental conditions cannot be controlled, surveillance testing of susceptible commodities will remain of vital importance. Increased production of cereals will be needed in the future to satisfy growing food demand in developing countries and feed demand in the newly industrializing countries. Under these circumstances, occurrence of mycotoxins in agricultural commodities will continue to remain on the health and economic policy agenda. The scope of the mycotoxin problem is readily understood when it is appreciated that there are many thousand secondary fungal metabolites, the vast majority of which have not been tested for toxicity or associated with disease outbreaks. Many fungi produce several mycotoxins simultaneously, especially *Fusarium* species. Co-occurrence of mycotoxins is of special concern, for instance, in the case of FUM (cancer promoter) and AFT (a potent human carcinogen) where a complimentary toxicity mechanism of action occurs. In developing countries it is likely that consumers will be confronted with a diet that contains a low level of toxin and in many cases, there may be other toxins present. 

Implicit with these conclusions are the existence of syndromes of apparently unknown etiology and epidemiology that may involve mycotoxins and the difficulty of establishing "no effect" levels for mycotoxins.
